# Deep convolutional architecture‐based hybrid learning for sleep arousal events detection through single‐lead EEG signals

**DOI:** 10.1002/brb3.3028

**Published:** 2023-05-18

**Authors:** Andia Foroughi, Fardad Farokhi, Fereidoun Nowshiravan Rahatabad, Alireza Kashaninia

**Affiliations:** ^1^ Department of Biomedical Engineering, Central Tehran Branch Islamic Azad University Tehran Iran; ^2^ Department of Biomedical Engineering, Science and Research Branch Islamic Azad University Tehran Iran; ^3^ Department of Electrical Engineering, Central Tehran Branch Islamic Azad University Tehran Iran

**Keywords:** EEG signal, deep learning, grey wolf optimization, Inception‐ResNet‐v2, sleep arousal, support vector machine

## Abstract

**Introduction:**

Detecting arousal events during sleep is a challenging, time‐consuming, and costly process that requires neurology knowledge. Even though similar automated systems detect sleep stages exclusively, early detection of sleep events can assist in identifying neuropathology progression.

**Methods:**

An efficient hybrid deep learning method to identify and evaluate arousal events is presented in this paper using only single‐lead electroencephalography (EEG) signals for the first time. Using the proposed architecture, which incorporates Inception‐ResNet‐v2 learning transfer models and optimized support vector machine (SVM) with the radial basis function (RBF) kernel, it is possible to classify with a minimum error level of less than 8%. In addition to maintaining accuracy, the Inception module and ResNet have led to significant reductions in computational complexity for the detection of arousal events in EEG signals. Moreover, in order to improve the classification performance of the SVM, the grey wolf algorithm (GWO) has optimized its kernel parameters.

**Results:**

This method has been validated using pre‐processed samples from the 2018 Challenge Physiobank sleep dataset. In addition to reducing computational complexity, the results of this method show that different parts of feature extraction and classification are effective at identifying sleep disorders. The proposed model detects sleep arousal events with an average accuracy of 93.82%. With the lead present in the identification, the method becomes less aggressive in recording people's EEG signals.

**Conclusion:**

According to this study, the suggested strategy is effective in detecting arousals in sleep disorder clinical trials and may be used in sleep disorder detection clinics.

## Graphical abstract

For the first time, this paper presents a hybrid deep learning method for identifying and evaluating arousal events using only single‐lead EEG signals. Utilizing the proposed architecture, which incorporates Inception‐ResNet‐v2 learning transfer models and optimized SVM‐RBF, a minimum error level of less than 8% can be achieved.

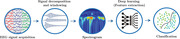

## 1 INTRODUCTION

Studies have shown that unfavorable sleep during rest negatively affects both work performance (Ting et al., 2014) and emotional well‐being (Galvão et al., 2021; Vandekerckhove & Yu‐lin, 2018). Arousals during sleep are a common indicator of poor sleep quality (Balakrishnan et al., 2006). A sudden change in electroencephalographic frequency occurring within 10 s of sleep onset is called electroencephalographic arousal by the American Academy of Sleep Medicine (AASM; Bonnet et al., 2007). Moreover, theta and alpha subbands, as well as frequencies over 16 Hz, have been observed to have changed. It is possible to determine the function of the subject's body during recording using a set of physiological signals known as polysomnography (PSG). These signals include electrooculography (EOG; Hasan et al., 2022), electrocardiography (ECG; Watling et al., 2021), electromyography (EMG), electroencephalography (EEG; Watling et al., 2021), respiration rate, respiratory flow, patient movement, and capnography.

Sleep arousal is a brief interruption of consciousness occurring during sleep caused by snoring or partial airway obstructions (Fernández‐Varela et al., 2017). Shortening of the sleep cycle occurs every 3 to 15 s after waking up. Most people do not notice when they are waking up in this condition; however, if they have been awake for more than 15 s, they may notice. The quality of human sleep decreases with frequent awakenings. The American Sleep Apnea Association reports that chronic sleepiness can be caused by as few as five arousals per hour.

In spite of the widespread problems associated with sleep irregularities, relatively little attention has been paid to automated detection and monitoring of apneas and arousal disorders (Zhang et al., 2022). Thus, sleep interruptions and daytime sleepiness are associated with non‐restorative sleep (Ghassemi et al., 2018).

As arousals during sleep are harder to detect using traditional methods, which makes research expensive, arousals during sleep are also less reliable for automatic detection than apneas (Engleman & Douglas, 2004). Early recognition of sleep hyperarousal is crucial to the diagnosis and treatment of sleep disorders (Fonod, 2022). It is possible to reduce the likelihood of sequelae, such as changes in blood pressure and cardiovascular disease, if the condition is detected early. Most state‐of‐the‐art arousal detection methods use multi‐channel recordings of PSG signals. Sleep experts visually grade 30‐s segments of PSG recordings according to standards established by the AASM (Berry et al., 2017). As a result of the large amount of data that must be analyzed, the process takes a considerable amount of time and effort. The sleep recording was recorded at 200 Hz and sampled 75 million times over an 8‐h period. Sleep recordings on this device can be manually recorded for hours. It is therefore necessary to create more efficient and consistent processes to meet the inter‐rater consensus of about 80% for the AASM standard (Altevogt & Colten, 2006). As time passes, the data record the patient's sleep and wakefulness phases, which can be analyzed by experts later. Despite their usefulness for analysis, PSG signals are both time‐consuming and difficult to collect. In addition, they require a lot of connections with tactile sensors, which can be uncomfortable for the subject and can alter the outcomes.

Previous research has attempted, with varied degrees of success, to accomplish automated arousal detection based on physiological markers. Despite the fact that the typical method shows that at least one EEG channel is beneficial, a number of authors have proposed utilizing alternative physiological signals. In the interim, the difficulties associated with employing a semi‐automated method to detect and assess arousal in EEG signals have been examined. Since previous studies used a variety of methodologies to diagnose various sleep disorders, developing a robust and objective approach is hard due to the wide variety of methodologies used. Moreover, the absence of a standardized threshold for the identification of sleep arousal is one of the primary difficulties in sleep arousal diagnosis. In this manner, it is necessary to overcome two major challenges that have not received much consideration in earlier automated techniques. In the first place, prior methods were unable to identify arousal issues that are associated with certain progressive brain diseases and their adverse effects on normal sleep. Second, in previous automated approaches, the uncertainty in recognizing arousal disorders is not addressed, causing neurologists to make an incorrect determination.

Automated sleep arousal detectors can be developed using a single‐lead EEG signal obtained painlessly (Johnstone et al., 2012). Sleep arousal information is extracted from single‐lead EEG data using an ensemble learning framework, which includes frequency domain, time domain, and expert‐described statistics. To classify and extract features, we implemented a hybrid learning framework combining an improved SVM with a 2D convolutional neural network (CNN). Prior to entering the convolution structure, the power spectrum of the signal is built in order to serve as a 2D input for the deep learning structure. The suggested strategy has satisfactory accuracy (92.14%), specificity (93.78%), and sensitivity (92.28%), compared to other similar models. This method combines deep learning and machine learning, which makes it different from traditional learning. The ResNet and Inception architectures are combined to create a deep learning architecture. Due to its robustness, speed, and statistical interpretation, a physician specializing in sleep disorders can better understand its structure. As a result of our study, we have made the following major contributions:

1. Understanding how to identify sleep hyperarousal is imperative for the early diagnosis and treatment of sleep disorders. Thus, we provide an effective and successful hybrid deep learning system for recognizing and rating arousal events from single‐lead EEG data in this study.
2. Less than 8% error rates are achieved when using the proposed architecture, which combines Inception‐ResNet‐v2 learning transfer models and optimized SVM with the RBF kernel. The grey wolf algorithm (GWO) has enhanced its kernel settings to increase SVM's performance in terms of classification.
3. The Inception module and ResNet have drastically lowered the computational complexity of recognizing arousal events in EEG data.
4. Due to the method's ability to be implemented and having a suitable output for various EEG signals, sleep disorder clinics can use it to diagnose sleep disorders efficiently.

Here are the other sections of this article: Section 2 discusses relevant literature. Sections 3 and 4 present the suggested method and experimental results. Section 4 discusses the results that prove the proposed sleep arousal detector. The conclusion of the research is presented in Section 5.

## 2 RELATED WORK

A number of works have been conducted in recent years on the use of EEG signals to recognize sleep arousal incidents in order to detect sleep disorders (Chien et al., 2021; Cho et al., 2007, 2006; Fonod, 2022; Liang et al., 2015; Olesen et al., 2020; Ugur & Erdamar, 2019). It has been suggested in the study by Cho et al. (2006) that a single‐lead EEG with time‐frequency analysis and an SVM classifier be used in order to determine arousal (C3‐A2). The test sets had values of 75.26 and 93.08%, while the training sets had values of 87.92 and 95.56%. There were nine individuals who participated in the study who had sleep‐disordered breathing.

Overfitting may cause these algorithms to contain errors that are caused by the way that they are designed. There have also been studies where the intensity of the EEG frequency band has been altered (Cho et al., 2007) in order to improve the classification of subjects. Compared to their previous study, this study showed lower sensitivity and specificity, respectively, at 79.06% and 89.95%. A single hidden layer feedforward neural network was trained using a novel curious extreme learning machine strategy by Liang et al. (2015) utilizing a single hidden layer feedforward neural network. Based on the signals derived from the central channels of the EEG, a total of 22 features were identified. It is significant to know that although they obtained accuracy rates of 83% and 79%, and an average area under the receiver operating characteristic curve (AUROC) of approximately 78.3%, their dataset is not accessible to the public. The total number of valid data pairs was 7680, of which only 144 were found to be valid. There are many arousal detection labels available for them (i.e., labels for arousal detection). A single‐channel EEG signal is processed in the study by Ugur and Erdamar (2019) using continuous wavelet transform (CWT), and then from the scalogram, two features—magnitude and square of the CWT—are extracted from it. In order to assess the presence of arousal in the brain signal, an SVM classifier is employed to automatically assess the presence of arousal. Prior to this study, it was stated that all three metrics (specificity, sensitivity, and accuracy) were greater than 94%, but the results should not be considered reliable because only EEG data from five patients was used for the calculation. Compatibility may change over time as more patient information becomes available, so it is crucial to recognize this fact.

It has been proposed by Olesen et al. (2020) that a deep transfer learning approach can improve single‐lead EEG arousal recognition by building a structure on multi‐channel PSG information, which is then shifted to a target region of single‐lead EEG signals in order to enhance single‐lead EEG arousal recognition. As a result of combining the principles of attention and fine‐tuning, it has been established that the differences between multi‐channel and single‐lead EEG PSG are negligible when compared to each other. Accordingly, the sensitivity and accuracy of the research are 71.0% and 67.0%, respectively, as a result of this study. For this form of transfer learning to work, it is necessary to eliminate the mismatch between the domains of the source and the target. It has been reported that a number of works have analyzed multiple input channels for the detection of arousal among the 13 different forms of raw PSG signals.

Chien et al. (2021), based on the meta‐classifier consisting of four submodels, constructed a sleep‐arousal detector using the PhysioNet dataset containing 994 PSGs. The classifier included 1D CNNs, recurrent neural networks (RNNs), merged CNNs and RNNs, and random forests (RFs). Based on AUROC, accuracy, sensitivity, and specificity, stacking ensemble learning outperformed a single submodel by 84.01%, 82.63%, 84.64%, and 80.10%, respectively.

Using artificial neural networks and two EEG channels and one EMG input, Alvarez Estévez and Moret‐Bonillo (2011) were able to detect EEG‐evoked events with the help of two EEG channels. In addition to their research, 39 EEG signal feature developments and four background components were analyzed as part of their analysis. According to the results of the work, the AUROC, specificity, and sensitivity were, respectively, 86%, 76%, and 81.10%. According to Liu et al. (2020), eight typical PSG signal pathways have been outlined. With the help of a deep learning architecture, CNN was provided with preprocessed signals for the eight channels. This was so that each CNN could make decisions independently of the others. In order to calculate the AUROC, they used the RF method and these previous choices, which yielded a value of 95.30 %. Based on PSG data as input, Wickramaratne and Shaad (2020) constructed sequence‐by‐sequence deep networks utilizing two‐way exponential linear units and long short‐term memory (LSTM) networks. It should be noted that each of the three models was based on a bidirectional LSTM (bi‐LSTM) network that was capable of receiving a variety of input signals. Using an equally weighted average approach to integrate the three models, the final classification result was determined by integrating the results of all three models. The estimated AUROC criteria were 95%.

A feature matrix was developed based on the submission of PSG inputs in terms of leg movement, EMG, EEG, breathing, and heart rate (Shahrbabaki et al., 2015). For the *k*‐nearest neighbor classifier, the average sensitivity, specificity, and accuracy for identifying sleep arousal events were 79%, 95%, and 93%, respectively. In order to establish the mean performance, however, only nine cases were used to establish the mean performance. According to the results, the lowest results were 67.50% sensitivity, 97.10% specificity, and 86.50% accuracy. Identification of sleep arousal models based on wide PSG signals may be hindered by methods that rely on broad PSG signals.

In Zhou and Guangxin et al. (2020), the authors selected six input channels after analyzing each PSG signal. Essentially, the EEG signal is made up of two elements. An architecture for deep learning was developed based on a ResNet network and a multi‐head situational attention system that relies on a ResNet neural network. Despite the fact that the overall AUROC was 84.88%, the AUROC for a single channel of the EEG was less than 76.20%. Using RF classification, Subramanian et al. (2018) reported an AUROC of 84.70 % with 27 features, including 14 EEG‐EOG, 7 ECG, and 6 EMG. A 13‐layer CNN and 13 raw PSG signals were used by the authors in Shen (2018) to obtain a 48.6% AUROC. The results of their study indicate that certain expert‐identified traits are likely to contribute to improved categorization outcomes. On the other hand, the area under precision‐recall curve (AUPRC) was 42.00% when the authors implemented a three‐layer neural network with 68 features from a matrix of 6821 features, while the AUPRC was 42.00% with a two‐layer neural network.

In Warrick and Nabhan Homsi (2018), the authors suggested that raw PSG signals should be transformed using the scatter transform. A three‐layer LSTM network was constructed with the help of 36 coefficients for each signal in order to construct a sequence learning machine. A batch normalization block was included in order to improve training outcomes for each layer; this resulted in an increase in the AUROC of 88.0%. A further investigation of this study employing a bi‐LSTM in order to enhance performance is described in Warrick et al. (2019). An analysis was carried out by the authors of Howe‐Patterson et al. (2018) in which multiple dense CNNs were connected to LSTM networks. This produced a dense recurrent CNN that incorporated 12 out of 13 PSG signals but did not incorporate ECG signals. As a result of this study, the AUROC was determined to be 93.10%.

A deep learning method for segmenting sleep arousal regions based on PSG signals was presented by Li and Guan (2021). Karimi and Asl (2021) automatically analyzed PSG recordings for non‐apnea sleep arousal zones. By using PSG signals, independent, sensor‐based features are generated in the time and frequency domains. Consensus techniques and group strategies have been employed to select a subset of features. The PhysioNet Challenge 2018 training set, consisting of 994 individuals, was used to validate and evaluate their suggested method. In 192 subjects, an AUROC score of 0.927 was obtained.

In view of the large variety of methodologies that are employed across past studies, it is impossible to compare the results of past studies in an exact and objective way. As explained in Chien et al. (2021 and Liang et al. (2015), the analyzed datasets, just like the physiological signals and performance indicators, are exclusive to individual researchers or are inaccessible to the general public. One of the fundamental problems with sleep arousal detection is the lack of a reliable threshold for recognizing sleep arousal.

## 3 PROPOSED MODEL

**FIGURE 1:**
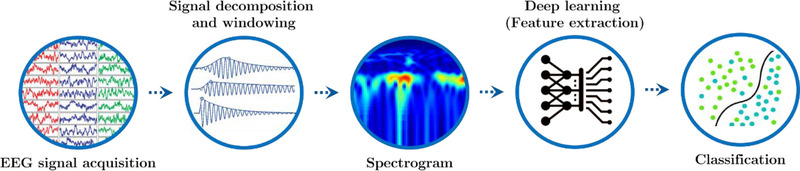
Implementation steps of the proposed method. EEG, electroencephalography.

As shown in Figure 1, the algorithm consists of three steps: windowing, feature extraction from deep learning structures, and classification.

### 3.1 Signal decomposition

High‐ and low‐frequency parts of EEG data are analyzed using discrete wavelet transforms, which require less processing time than wavelet transforms (WT). The gamma band (*γ*), beta band (*β*), alpha band (*α*), theta band (*θ*), and delta band (*δ*) can be used to categorize EEG background waveforms (Al‐Kadi et al., 2014; Al‐Qazzaz et al., 2015). It is also possible to obtain useful information from EEG background waves. With its numerous resolutions, WT is a new technique for time‐frequency analysis. Both temporal and frequency localizations are possible with WT. Band‐pass filters separate signals into various frequency bands. Sleep problem sufferers may experience similar effects from EEG data extraction patterns as non‐arousal participants. It would be very useful to segment the EEG data into various subbands and frequencies since both types of signals produce similar patterns. The EEG signal from subjects needs to be decomposed into subbands, and these rhythms are based on different frequencies of the separation format signal. Gamma bands are defined as higher than 30 Hz, beta bands as higher than 12 Hz, alpha bands as higher than 12 Hz, theta bands as higher than 4 Hz, and delta bands as lower than 4 Hz. Using a classification algorithm and localized wavelet filter bank characteristics, we can detect anomalous arousal conditions in participant EEGs. For signal dimension reduction, the EEG signal is first converted into wavelet coefficients using a wavelet decomposition algorithm employing DB3 (Level 3).

### 3.2 Signal windowing

When processing non‐stationary physiological signals, windowing is essential for reducing signal complexity. The EEG signal is windowed before features are retrieved from each produced frame with a specific amount of overlap. Because the duration of the EEG signal windowing process varies from state to state, selecting the duration and degree of overlap between successive frames may be challenging. For the EEG data to be split into equal time intervals, all frames must have the same length and overlap with the frames before and after them.

### 3.3 Spectrogram

Using windowed Fourier transform expansions or short‐time Fourier transforms (STFTs), signal power spectrum and framing can be created. The moving window function *g*(*t*) is applied to the signal *x*(*t*) at time *τ*. For each specific time *τ*, *x*(*t*) within the window is transformed using a finite time Fourier transform. Alternately, the window is shifted by τ in the direction of time and the Fourier transform is applied. As a result of this alternating process, the Fourier transform of the whole signal is calculated, while the signal part inside the window is treated as static. STFT transfers a signal in the time range to 2D time‐frequency display, and the changes in the frequency content of that signal are displayed in the window. As shown in (1), STFT can be defined as follows:

(1) $$STFT\left(\tau ,f\right)=\int x(t)g(t-\tau )\;e^{-2j\pi ft}dt.$$

STFT has a limitation, which is that if the window size is chosen once, the frequency‐time separation will remain constant throughout the frequency‐time plane even as *g*(*t*) changes. Because of this, choosing an appropriate window size in the STFT method will be challenging when both high‐frequency and low‐frequency components are present in the signal at the same time. Final accuracy also determines the number of windows produced. The pre‐training network can display the framed signals as a signal power spectrum (a signal power drawing based on the frequency component and the time component) for use in the classification stage. A rectangular window represents the signal power (i.e., energy per unit time) per unit frequency for a given signal. In a given frequency unit, the power spectrum represents a part of the signal power.

### 3.4 Feature extraction

Convolution filters are used in CNN networks to extract image features. Filters determine what features are extracted from an image based on their size. Before the introduction of Inception modules, network designers had to determine the size of the filters. The Inception family of architectures incorporates residual connections (instead of filter concatenation) into a convolutional neural architecture called Inception‐ResNet‐v2. Nevertheless, ResNet's structure improves differentiation between classes, resulting in a more robust set of features. However, with the increasing sampling rate, it is also possible that the previous layers will not maintain the appropriate features in some cases (especially when the feature mapping is thin to reduce processing time). There has already been evidence that this structure saves time and improves accuracy (Szegedy et al., 2017). There is a hybrid primitive module in Inception‐ResNet‐v2, which is inspired by ResNet's transfer learning model. Inception ResNet has two minor versions, version 1 and version 2. The modules A, B, and C of both subversions, as well as the reduction blocks, share the same structure. There is only a difference in the hyperparameter settings. In order to set hyperparameters precisely, many methods have been proposed.

**FIGURE 2:**
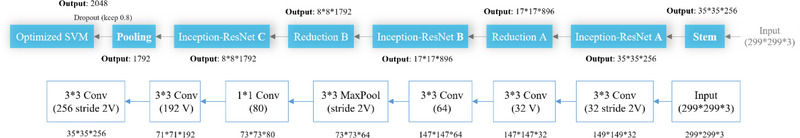
Inception‐ResNet‐v2 has been illustrated in its architecture, and the details of its structure are presented in Figure 3.

**FIGURE 3:**
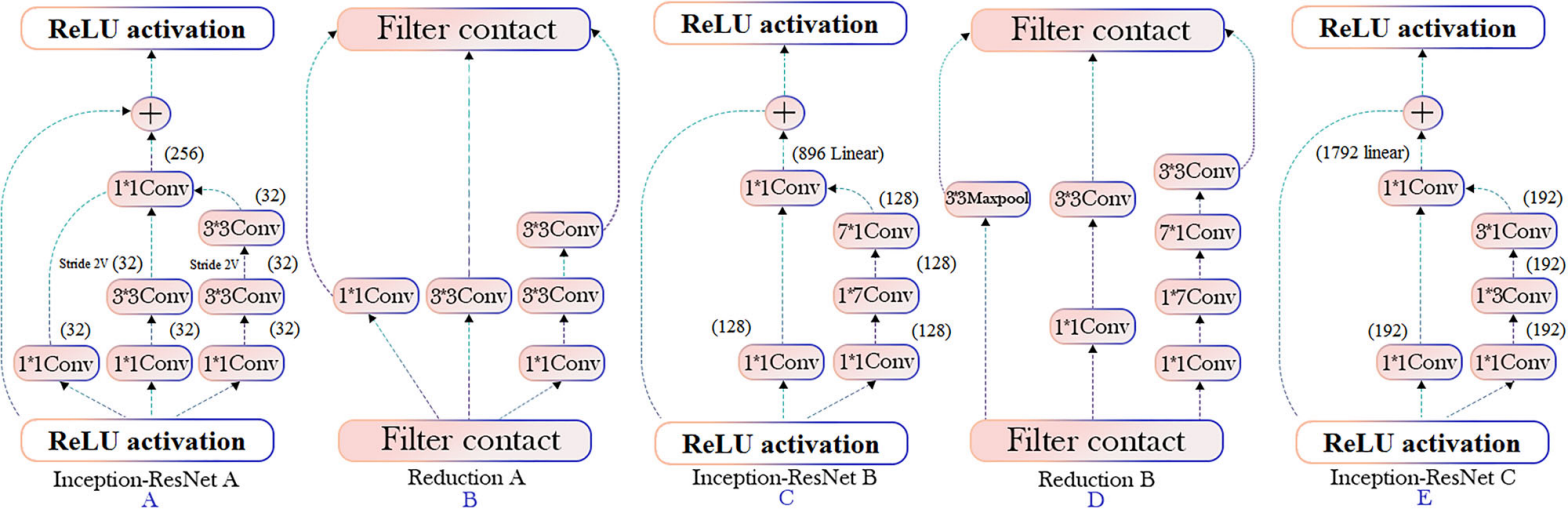
An outline of the design of the Inception‐Reduction‐Network A, B, C and Reduction‐Network A, B.

The residual connections are considered the output of the initial module's complexity operation. In order to add a residual to the study, the output and input after convolution must have the same dimensions. After the original convolutions, 1 × 1 convolutions are used to match the depth sizes (the depth increases after the convolution). It is depicted in Figures 2 and 3 that the combined structure of ResNet and Inception includes convolution, average pooling, maximum pooling, concatenation, drop‐out, fully connected layer, and Softmax. An image of Inception‐Resnet‐v1 and Inception‐Resnet‐v2 can be found in the above part of Figure 2. Although both networks have the same fundamental structure, their stems and inner modules differ. By contrast, the InceptionResnet‐v2 network stem is similar to the pure Inception‐v4 network. The interior modules are illustrated in Figure 3. S indicates the use of “same” padding, while V indicates the use of “valid” padding. Sizes to the left and right of each layer summarize its output form.

### 3.5 Classification

Using a categorization framework, we construct models that predict output characteristics based on input quality. By incorporating an appropriate strategy within the machine learning framework, SVM improves data classification.

SVMs are designed to reduce class separation by identifying the optimal hyperplane. Furthermore, the *Φ* kernel function detects highly complex inputs with wider coverage. With this kernel function, we can linearly separate data in more than two dimensions. Using *D* features and a single training dataset, we can create a function that translates input into output:

(2) $$f\left(x,w\right)=\;w^{T}x+b.$$

Function *f* can only be obtained by minimizing the values of *w* and *b* in this equation:

(3) $$R\left(C\right)=\frac{1}{2}{\left\|w\right\|}^{2}+C\frac{1}{l}\sum_{i=1}^{l}L_{\varepsilon }\left(y_{i},f_{i}\left(x,w\right)\right).$$

We will assume that the *C* (a soft margin parameter) variable is a fixed parameter that can be adjusted by the user. Due to the absence of the variable *ε*, this parameter is aimed at stabilizing and modifying penalty weights while also boosting discrimination capacity. As a result, the *L_ε_* function is presented in the following manner:

(4) $${\left|y-f\left(x,w\right)\right|}_{\varepsilon }=\left\{\begin{array}{cc} 0 & \left|y-f\left(x,w\right)\right|\leq \varepsilon \\ \left|y-f\left(x,w\right)\right|-\varepsilon & \mathrm{otherwise} \end{array}.\right.$$

As a maximum of the previous equation, the following one is written:

(5) $$\begin{array}{ccc} L_{P}\left(a_{i},a_{i}^{*}\right)= & & -\frac{1}{2}\sum_{j=1}^{l}\left(a_{i}-a_{i}^{*}\right)\left(a_{j}-a_{j}^{*}\right)x_{i}^{T}x_{j}\varepsilon \sum_{i=1}^{l}\left(a_{i}+a_{i}^{*}\right)\\ & & +\sum_{i=1}^{l}\left(a_{i}-a_{i}^{*}\right)y_{i}, \end{array}$$

where Equation (5) is used to define conditions:

(6) $$Condition:\left\{\begin{array}{c} \sum_{j=1}^{l}\left(a_{i}-a_{i}^{*}\right)=0\\ 0\leq a_{i}\leq C,i=1,\text{…},l\\ 0\leq a_{i}^{*}\leq C,i=1,\text{…},l \end{array}\right..$$

By analyzing the mentioned equations, the SVM function, that is, *f* in Equation (2), can be achieved utilizing the kernel function:

(7) $$f\left(x,w\right)=w_{0}^{T}x+b=\sum_{j=1}^{l}\left(a_{i}-a_{i}^{*}\right)x_{i}^{T}x+b.$$

In the first half of the experiment, classification is applied to training data. SVM classifier with RBF kernel initializes *γ* (the inverse of standard deviation) and *C* randomly. There is a difference in the speed at which each of these qualities changes. Accordingly, kernel RBF can be described as (8) for support vectors:

(8) $$K\left(x,x_{j}\right)=\exp(-{\left\|x-x_{j}\right\|}^{2}\times {\left(2\sigma^{2}\right)}^{-1}.$$

Sigma (*σ*) specifies the level of non‐linearity included in the model and is comparable to the kernel function's bandwidth. When sigma is small, the decision boundary will be highly non‐linear. By contrast, a linear decision boundary is more prevalent when sigma is large. Based on the training data, the GWO method (Mirjalili et al., 2014) selects the structure with the least amount of error. GWO considers alpha the best solution, beta the second‐best solution, and delta the third‐best solution. All other solutions are considered omegas. In the GWO algorithm, hunting is driven by α, β, and *δ*. The solution ω follows these three wolves. When the prey stops moving and is surrounded by wolves, the α wolf leads the attack. This process is modeled by reducing the vector “*x*.” The vector of coefficients of “*X*” decreases as “*x*” vector decreases since “*X*” is a random vector in the interval −2x to 2x. If |X| < 1, the α wolf will approach the prey (and other wolves) and if |X| > 1, the wolf will distance itself from the prey (and other wolves). According to the GWO, all wolves must update their positions based on the positions of *α*, *β*, and *δ* wolves. Alphas usually lead hunting operations. Hunting may occasionally be conducted by beta and delta wolves. Using a mathematical model of grey wolf hunting behavior, we hypothesized that alpha, beta, and delta have a better understanding of possible prey locations. According to the following equations, the other agent is required to update its positions based on the positions of the three best search agents. Until the end of the algorithm, the top three answers are selected as *α*, *β*, and *δ*.

- In each iteration, the three best answers (*α*, *β*, and *δ* wolves) estimate the position of the prey using the following relationship:
- Each time the position of *α*, *β*, and *δ* wolves is determined, the rest of the answers are updated accordingly.
- Vector *x* (and consequently *X*) and other related vectors are updated with each iteration.
- As a result of the iterations, the alpha wolf position is introduced as the optimal position.

By using this method, convergence toward the optimum response is accelerated and fine‐tuned. To evaluate the classification structure, the average accuracy, and the most effective matching parameters, the optimization procedure is repeated five times. The accuracy of the network plays an influential role in determining an appropriate match. Searching for the GWO algorithm for global optimization can be assisted by calculating the optimal values of the RBF kernel.

Based on the fitting of extracted features from spectrogram images, we optimize the parameters of RBF kernels. GWO's cost function is computed on the classification error of each classifier each time the algorithm is called in the optimization loop. The initial population of wolves is selected based on characteristics with high influence (alpha wolf), high influence (beta wolf), medium influence (delta wolf), and finally low influence (omega wolf). To select the most optimal parameters in the RBF kernel, meta‐heuristic algorithms repeat generation until there is no convergence boundary or until the number of iterations is limited. The algorithm can find the global optimum faster than other similar methods because of the presence of various types of wolves in the search of the solution space.

## 4 EXPERIMENTAL RESULTS

The results and interpretations are presented in this section. First, physiological signals are discussed.

### 4.1 Physiological signals

PhysioNet was used to validate the suggested technique as part of the Challenge 2018 project (Ghassemi et al., 2018). Neurological specialists interpreted physiological signals including ECG, EEG, abdominal (ABD), arterial oxygen saturation (SaO_2_), chest, and AIRFLOW from a large number of subjects in both data and distinct time periods. In the dataset, 1983 people were observed in a sleep laboratory at Massachusetts General Hospital (MGH) to diagnose sleep disorders. The essential EEG signal captures arousal events as demonstrated by the dataset. Similar to an EEG signal, a cardiac signal's duration is measured in minutes and seconds. During the incident, the amount of dissolved oxygen in the blood was also assessed. In general, blood contains the lowest levels of dissolved oxygen. Based on the type of occurrence or sleep state, each record includes all manual registration remarks from the specialist's files. The Sleep Division of the Sleep Lab at MGH, the Computational Clinical Neurophysiology Laboratory, and the Clinical Data Animation Center submitted 1983 PSG recordings. The PSG was captured according to AASM specifications. In accordance with the International 10/20 System, EEGs were recorded at O2‐M1, O1‐M1, C4‐M1, C3‐M2, F4‐M1, and F3‐M2. Left‐sided EOG was performed (EEG and EOG refer to the contralateral ear lobe); 200 Hz sampling frequency was used for all signals with the exception of the SaO_2_. In the training set, the arousal labels for 994 recordings were displayed, whereas in the test set, 989 labels were concealed. The examination and training sets were distinct. In the training set, arousal labels were selected by specialists and neurologists.

According to the full dataset, arousal or excitatory regions are classified as “1” in around 4% of instances, non‐excited regions are classified as “0” in 80% of instances, and undefined regions are classified as “−1.” Arousal zones have a minimum duration of around 30 s and a maximum duration of nearly 4 min, based on the data. Furthermore, most of these non‐apnea arousal zones are in Stages 1 and 2, with a few in Stage 3. The distribution of subject arousal is shown in Table 1. In addition, the values mentioned in the table are not based on the sample but on the basis of the event.

**TABLE 1:** The distribution of subject arousal is shown in this table.

**Type of arousal events**	**No. target arousals**
Noise	1
Cheyne‐stokes breathing	3
Hypoventilation	4
Partial airway obstruction	11
Bruxism	30
Snoring	28
Periodic leg movement	36
Spontaneous	70
Mixed apnea	2641
Central apnea	22,763
Obstructive apnea	32,547
Respiratory effort‐related arousals	43,822
Hypopnea	56,936
Number of non‐target arousals	682,526

### 4.2 Setting

A 64‐bit operating system and 4GB of RAM were used for Intel (R), Core (TM), and Core i7 processors. Analyses of quantitative and qualitative results were performed using MATLAB programming tools (2022a version). In order to detect sleep events based on the duration of certain signals, EEG signals were synchronized with the sleep phases of each subject.

**FIGURE 4:**
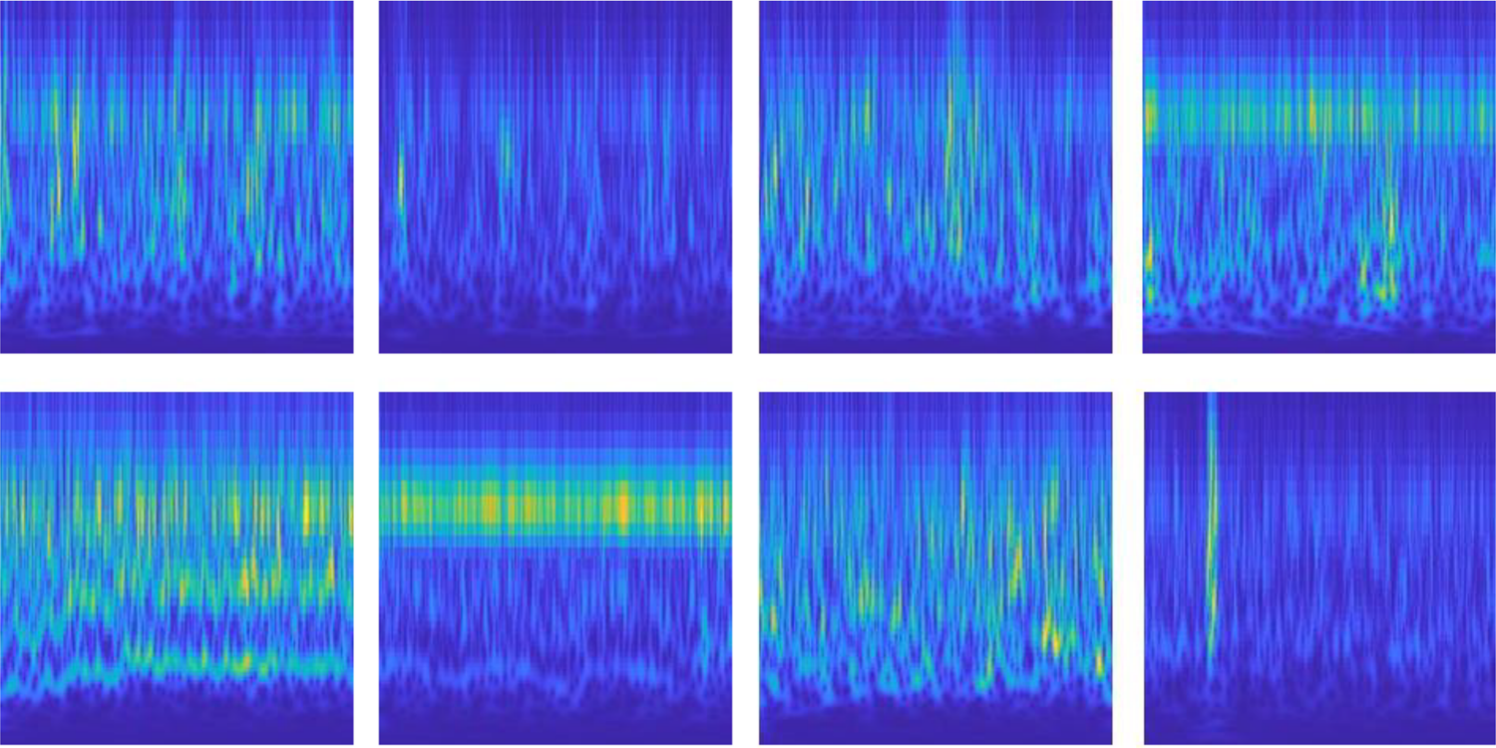
Spectrogram images of the signals in which there are no indications of arousal have been displayed in the first row (the first four), while the spectrogram images of the signals in which there is an indication of arousal have been shown in the second row (the second four).

It is a two‐class problem to classify arousal episodes from EEG data containing many subbands. To feed the deep learning model with spectrogram images of each segment, EEG data were divided into discrete windows (1‐s intervals) and deep sleep bands. In each spectrogram image, 1536 features are extracted from the deep structure of the last layer before entering the prediction section and the fully connected layer. An example of a spectrogram of segmented signals from EEG signals with and without arousal is shown in Figure 4.

Additionally, batch normalization, scaling, addition, ReLU, 2D global average pooling, and depth concatenation layers are also used to make up the Inception‐ResNet‐V2 compound. Spectrograms with a resolution of 299 by 299 pixels are fed into the structure. Initially, Inception‐ResNet‐V2 had a learning rate of 0.001. Between 1000 and 2000 epochs, the utilized model improved its learning performance. A stochastic gradient descent and root mean square propagation were also used to tune the Inception‐ResNet‐V2 architecture. To use the Inception‐ResNet‐V2 model in production, it required 8–12 h of training on a single CPU. Different types of structures were also obtained from SVM, each having a different output with a maximum standard deviation of 3.1% depending on the kernel parameters. A certain number of iterations were used to search for optimal parameters in hyperplanes obtained through GWO in offline mode for use in online mode. In addition, the initial population of wolves, the number of repetitions in the optimization strategy, the number of repetitions in execution, the agent parameter for the optimization approach, the first mutation parameter, and the second mutation parameter are equal to 20, 100, 10, 2.5, 0.25, and 0.5, respectively. According to research (Mirjalili et al., 2014), these parameters were chosen based on faster convergence results as well as values suggested in research. Furthermore, the search intervals for selecting *C* and *γ* parameters were set to 1 to 50 and 1 to 30, respectively.

### 4.3 Evaluations

The confusion matrix and ROC curve are employed to assess the efficiency of the suggested model. In the confusion matrix, there are four states of true positives (TP), true negatives (TN), false positives (FP), and false negatives (FN) employed to analyze the classification and recognition of non‐arousal events and arousal events. Using these definitions, it is possible to estimate accuracy, sensitivity, and specificity using Equations (9) to (11):

(9) $$Accuracy=\left(\frac{N_{TP}+N_{TN}}{N_{TP}+N_{FN}+N_{TN}+N_{FP}}\right),$$

(10) $$\mathrm{Sensitivity}=\left(\frac{N_{TP}}{N_{TP}+N_{FN}}\right),$$

(11) $$Specificity=\left(\frac{N_{TN}}{N_{TN}+N_{FP}}\right).$$

**FIGURE 5:**
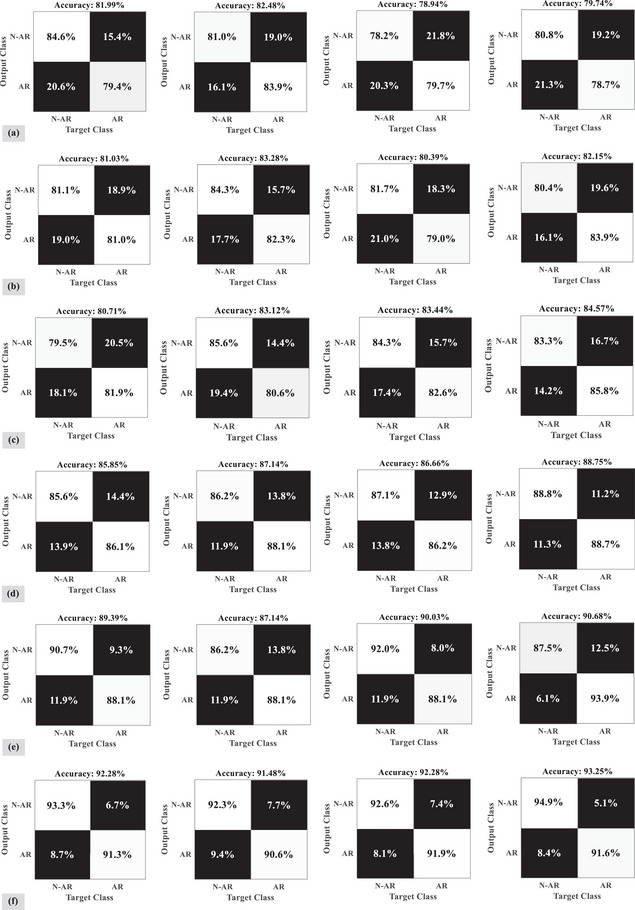
The algorithm is implemented in four categories of folds for channels (a) o2‐M1, (b) c4‐M1, (c) c3‐M2, (d) f3‐M2, (e) f4‐M1, and (f) o1‐M2. AR, arousal; N‐AR, non‐arousal.

As shown in Figure 5, confusion matrices represent the performance of the suggested strategy in identifying arousal based on the different channels. These figures depict the outcomes of applying the model to the analysis of EEG channels, respectively, a (o2‐M1), b (c4‐M1), c (c3‐M2), d (f3‐M2), e (f4‐M1), and f(o1‐M2) for the posterior, central, central, frontal, and posterior parts of the brain. Based on these channels, accuracy ranged between 80% and 93% on average, with (o1‐M2) having the highest accuracy rate.

**FIGURE 6:**
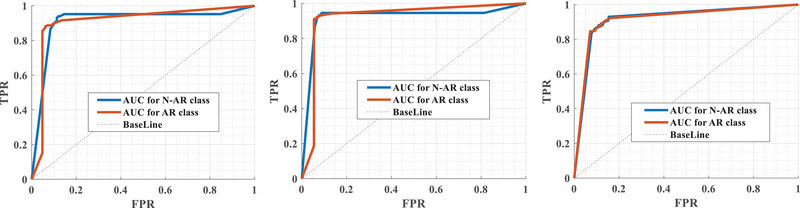
Applying the proposed model to different test signals received from the same channel and drawing receiver operating characteristic curves. There are separate curves in each of the three figures indicating the presence or absence of arousal. AR, arousal; N‐AR, non‐arousal.

Channel (o2‐M1) has the lowest level of accuracy in classifying arousal from an EEG signal. Additionally, based on the evidence, the posterior and central parts of the brain may be able to provide more information about arousal. Moreover, each figure shows four random folds out of 10 folds and the final accuracy. In the ROC, the receiver agent describes the uncertainty problem. Figure 6 shows that the average error of the suggested algorithm for detecting arousal based on EEG recordings is generally lower than other methods, and the area under the measurement error curve can be used to quantify this issue according to (12).

(12) $$\;A_{z}=\frac{1}{N_{TP}.N_{TN}}\sum_{v=1}^{N_{TP}}\sum_{nv=1}^{N_{TN}}I(N_{TP_{v}},N_{TN_{nv}}).$$

The parameters of *A_z_* (area under curve) are defined by Equations (9)–(11).

### 4.4 Discussion

**FIGURE 7:**
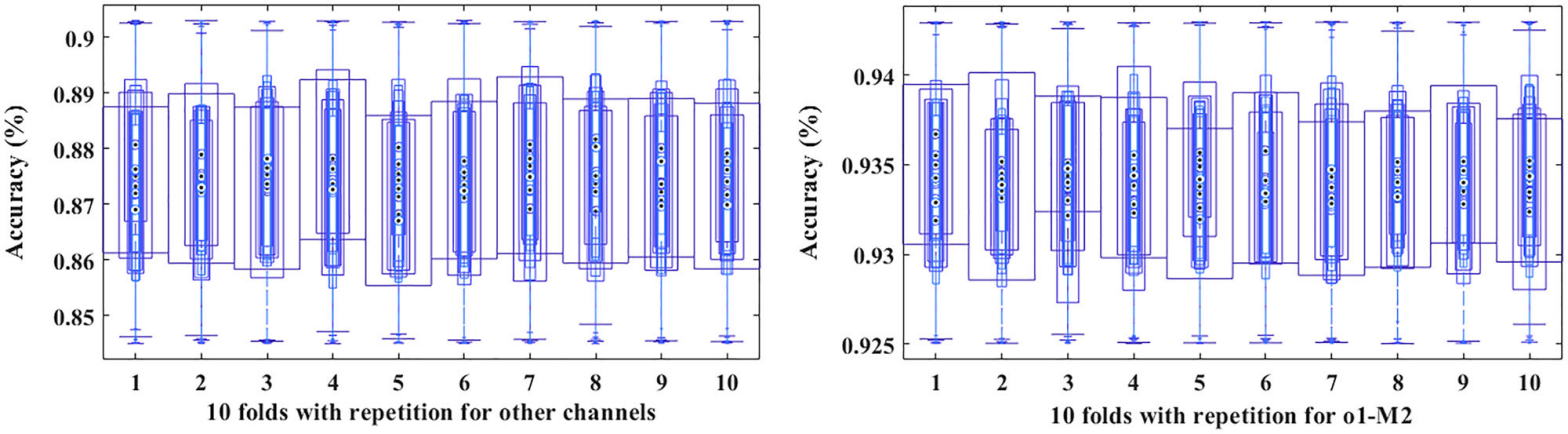
According to box plots, the o1‐M1 channel is more effective than the other channels in detecting arousal events in several repetitions of the algorithm.

**FIGURE 8:**
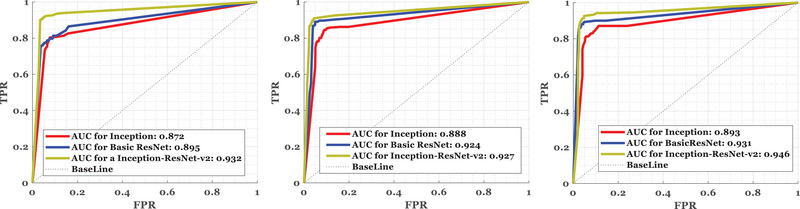
According to the repeatability of the Inception‐ResNet‐v2 algorithm in feature extraction compared to two similar algorithms, Inception and Basic ResNet, it has a higher AUC. In light of these findings, it can be concluded that the method is robust at identifying arousal after a number of iterations.

A five‐repetition test to assess accuracy in detecting arousal disorders through the o1‐M2 channel and other channels shows the differences that the proposed algorithm has created. A box plot showing the results of different signals, including arousal and non‐arousal signals, is depicted in Figure 7. In another experiment, the signals were subjected to noise conditions similar to motion noise from people. In two situations with low complexity and high complexity, the estimates were accepted. For the training and test data, the proposed method in Table 2 was considered considering low complexity (first part) and high complexity (second part). Based on the ROC curve shown in Figure 8, we compared similar models with the proposed model. The AUC amount for unseen signals in feature extraction has been compared for three models: Inception, Basic ResNet, and Inception‐ResNet‐v2 for arousal detection. As a result of its performance, Basic ResNet can be considered a suitable model for creating features obtained from the spectrogram image.

**TABLE 2:** Arousal detection has been done for the training and test signals in the presence of people's movement noise, and the output of the algorithm is examined in two situations of low and high complexity.

**Complexity**	**Training signals**			**Test signals**		
	**Accuracy**	**Sensitivity**	**Specificity**	**Accuracy**	**Sensitivity**	**Specificity**
Part 1	93.43	93.11	93.78	92.14	92.28	93.78
Part 2	89.62	90.07	89.44	89.37	89.11	89.54

It can be seen, however, that it improves accuracy somewhat when combined with the structure of Inception. As shown by the high AUC criterion, the model has performed well in generating both positive and negative errors, and its stability for AUC values indicates the method's robustness.

The current study assessed arousal with the only single‐lead recording of EEG signals occurring during sleep rather than previous studies that relied on PSG signals to detect arousal. Despite the fact that some arousal methods have relied less on physiological signals in recent years, some of them are computationally complex (Jabari et al., 2023). Some other methods have either used PSG signals or analyzed EEG signals without considering the elements underlying their relationship. To identify unique sleep events with greater than 90% accuracy using standard algorithms, precise data patterns must be extracted. According to our research, combining deep features with handcrafted features (Badiei et al., 2023) can help improve accurate estimation of arousal events. Table 3 compares excitation detection models based on metrics such as computational complexity and accuracy.

**TABLE 3:** This table compares excitation detection models based on metrics such as computational complexity and accuracy.

**Author**	**Signal**	**Accuracy**	**Computational complexity**	**Adv/Disadv**
Liang et al. (Liang et al., 2015)	EEG	87.00%	Moderate	Using the extreme learning machine/the existence of the challenge of overfitting and lack of generalizability
Ugur et al. (Ugur & Erdamar, 2019)	EEG	93.00%	High	Single channel and proper accuracy/little data and lack of generalizability
Chien et al. (Chien et al., 2021)	EEG	84%	High	Single channel and accuracy are good/all signals have been investigated and lack of generalizability
Liu et al. (Liu et al., 2020)	PSG	86%	High	Single channel and accuracy are good/all signals have been investigated and lack of generalizability
Guan and Li (Li & Guan, 2021)	PSG	92.80%	High	Appropriate accuracy/the use of all channels and signals, as well as existing challenges with overfitting and generalizability
Proposed method	EEG	93.82%	Moderate	Single channel and proper accuracy, only brain signals have been examined and can be generalized/accuracy can be improved

Abbreviations: EEG, electroencephalography; PSG, polysomnography.

## 5 CONCLUSION

This study describes a method for efficiently gathering and classifying relevant features from single‐lead EEG signals using optimal learning. We created a hybrid deep learning strategy based on single‐lead EEG sleep signals to identify arousal events. In addition, we planned to identify discrete occurrences of awakening and arousals that could result in progressive brain disease, an EEG signal anomaly that might be temporary. However, it gives essential information regarding the likelihood of developing multiple system atrophy, Parkinson's, and Alzheimer's disorders. These difficulties have been linked to these diseases, according to strong evidence. Based on GWO and SVM‐RBF, an optimal learning model was developed for describing arousal episodes. We constructed a discriminative technique for analyzing physiological signals by using Inception‐ResNet‐v2 and extensively extracted features. As a means of preventing a range of sleep‐related diseases, future studies could examine the relationship between ECG and EEG signals. In the future, we will focus on detecting arousals and estimating their intensity based on a small number of signal channels and subbands. Moreover, the authors will try to reduce the computational complexity and improve the accuracy.

## CONFLICT OF INTEREST STATEMENT

The authors declare no conflicts of interest.

### PEER REVIEW

The peer review history for this article is available at https://publons.com/publon/10.1002/brb3.3028.

## Data Availability

The data that support the findings of this study are openly available in “Physionet” at https://archive.physionet.org/physiobank/database/challenge/2018, reference number (Fernández‐Varela et al., 2017).
